# Protein metalation in a nutshell

**DOI:** 10.1002/1873-3468.14500

**Published:** 2022-09-26

**Authors:** Deenah Osman, Nigel J. Robinson

**Affiliations:** ^1^ Department of Biosciences University of Durham UK; ^2^ Department of Chemistry University of Durham UK

**Keywords:** cobalt, copper, iron, Irving–Williams series, magnesium, manganese, metal sensor, metalation, nickel, zinc

## Abstract

Metalation, the acquisition of metals by proteins, must avoid mis‐metalation with tighter binding metals. This is illustrated by four selected proteins that require different metals: all show similar ranked orders of affinity for bioavailable metals, as described in a universal affinity series (the Irving–Williams series). Crucially, cellular protein metalation occurs in competition with other metal binding sites. The strength of this competition defines the intracellular availability of each metal: its magnitude has been estimated by calibrating a cells' set of DNA‐binding, metal‐sensing, transcriptional regulators. This has established that metal availabilities (as free energies for forming metal complexes) are maintained to the inverse of the universal series. The tightest binding metals are least available. With these availabilities, correct metalation is achieved.

## Graphical abstract

Metalation of proteins is logical within defined intracellular metal availabilities. Mis‐metalation is avoided because weaker binding metals are more available than tighter binding ones.

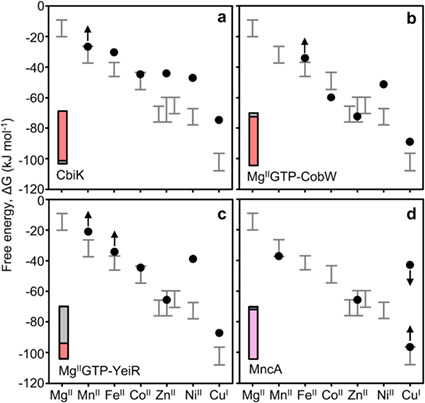

## Abbreviation

**SodA**, superoxide dismutase

## Metal affinities and mis‐metalation

**Fig. 1:**
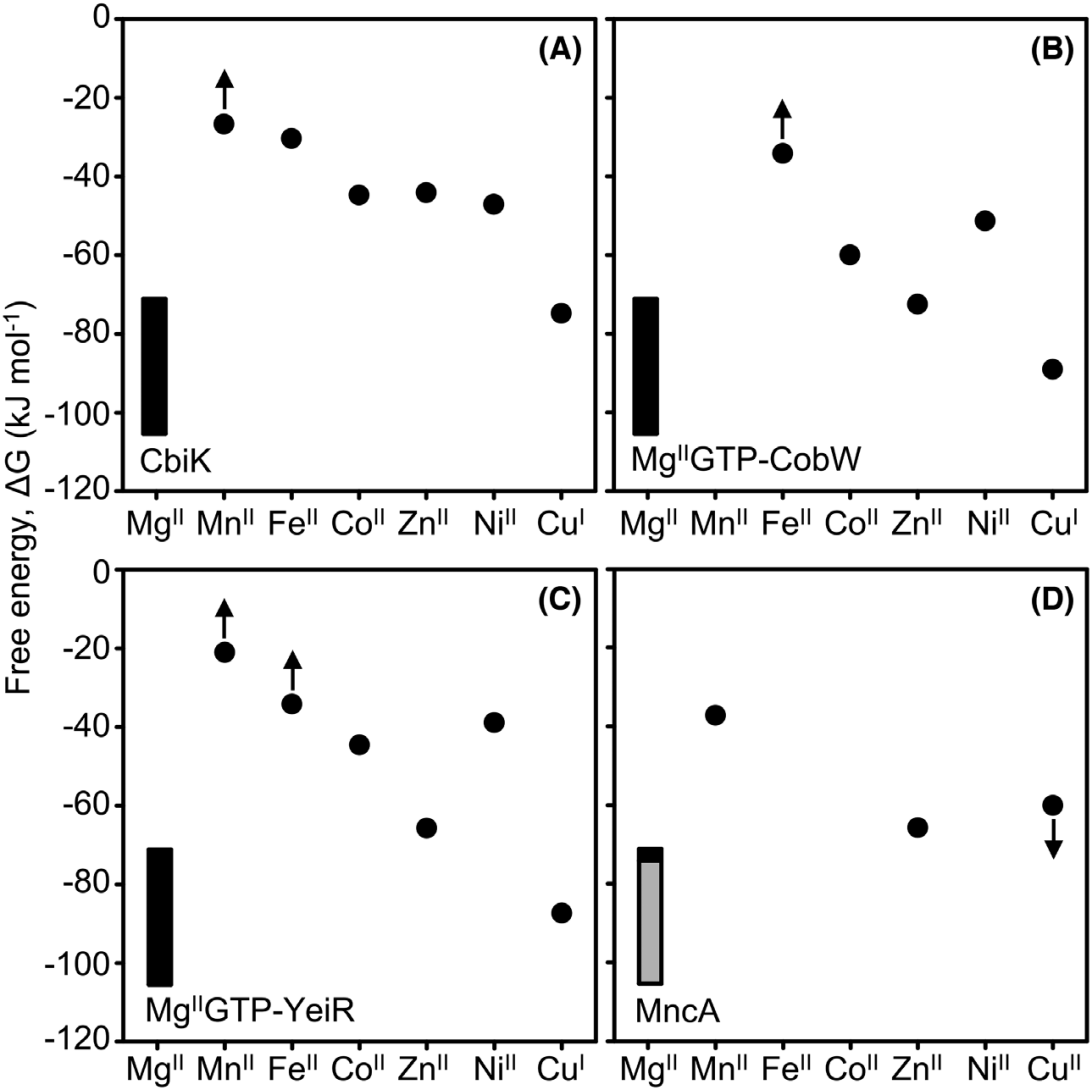
Metal binding to four proteins exemplifies the risk of mis‐metalation. Tightness of binding to the four proteins is shown for the available and exchangeable forms of metals in the cytosol (or Cu^II^ for periplasmic MncA). Values (black circles) are free energies for forming complexes calculated *via* the standard relationship ∆*G* = −*RT*ln*K*_A_ (∆*G* free energy change, *R* molar gas constant, *T* temperature in kelvin, *K*_A_ association constant). Note that values are logarithmically related to binding constants. The more negative the value the tighter the binding. Arrows indicate values that were at the minimum or maximum limits of the respective determinations of metal affinity. CbiK is a Co^II^ chelatase for vitamin B_12_ biosynthesis [3], Mg^II^GTP‐CobW is a Co^II^ metallochaperone from an alternative vitamin B_12_ biosynthetic pathway, Mg^II^GTP‐YeiR is analogous to Mg^II^GTP‐CobW but implicated in handling Zn^II^ [5], MncA is a Mn^II^ cupin [4]. Values for MncA are assigned based upon competition between metals (details noted in the text). Insets show percentage occupancies with copper (black) and Zn^II^ (grey) as a proportion of total metal occupancy. The correct metals are not the tightest binding metals and the orders of binding tend to follow the Irving–Williams series [6, 7].

Metalation is crucial for metalloproteins to achieve their proper enzymatic activity and/or structure. Mis‐metalation occurs in part because proteins are flexible and metal binding is non‐conservative: a wrong metal can use a sub‐set of ligands from the *bona fide* site, recruit additional adventitious ligands, and/or distort the native geometry. With such limited constraint, it is anticipated that most metalloproteins are at risk of mis‐metalation of their nascent binding sites. It is estimated that 47% of enzymes contain metals [1, 2]. How do cells overcome this pervasive challenge to enable enzymes to bind metal(s) with the correct chemical properties, and not simply those that bind most tightly? To exemplify the challenge of mis‐metalation, Fig. 1 shows how tightly essential metals bind to the four selected proteins: Namely, a chelatase (CbiK) that acquires Co^II^ for a molecular cofactor (vitamin B_12_), a Co^II^ metallochaperone (CobW), a homologous Zn^II^ delivery protein (YeiR) and a Mn^II^ enzyme that entraps metal during folding (MncA) [3, 4, 5]. The tightness of binding is represented as differences in free energies for forming the respective metal complexes. Affinities were measured for what is considered the exchangeable available form of each metal in the cytosol, that is divalent except for copper which is monovalent (albeit MncA ratios were estimated for Cu^II^ as described later) [6]. For all four proteins, the tightest binding metal is not the one required for activity (Fig. 1). The orders of binding follow, or tend towards, the Irving–Williams series: Mg^II^ < Mn^II^ < Fe^II^ < Co^II^ < Ni^II^ < Cu^II^ (Cu^I^) > Zn^II^, from weakest to tightest [6, 7]. If surplus metals were allowed to inter‐compete, three proteins would be mis‐metalated with Cu^I^, and one inferred to become mis‐metalated with Zn^II^ (represented as black and grey insets in Fig. 1).

### Examples of delivery proteins and the synthesis of molecular cofactors

Some proteins bind metals in pre‐assembled cofactors such as iron in haem or iron sulfur clusters, nickel in cofactor F_430_ or cobalt in vitamin B_12_, as examples. However, the correct metal must still partition onto the cofactor assembly pathway in the first place. CbiK is a chelatase that inserts Co^II^ into corrin in one of two pathways for the synthesis of vitamin B_12_ [8, 9, 10]. Fig. 1 shows that Cu^I^ binds tightly to CbiK, with Zn^II^ and Ni^II^ comparable to Co^II^, highlighting the question as to how tight‐binding non‐cognate metals are avoided.

Metals are supplied to some proteins by delivery pathways involving metallochaperones and here the final metalation step is aided by selective protein–protein interactions [11, 12, 13, 14]. CobW supplies Co^II^ to a different chelatase, not CbiK, in an alternative pathway for vitamin B_12_ biosynthesis [5, 8, 15]. CobW has a predicted GTPase domain and when bound to Mg^II^GTP, CobW forms a tight complex with Co^II^ but crucially it forms an even tighter complex with Cu^I^ [5]. As an aside, binding of Ni^II^ to Mg^II^GTP‐CobW is relatively weak, departing from the Irving–Williams series (Fig. 1). This is probably because association with Mg^II^GTP pre‐organises the Co^II^ site of CobW into a tetrahedral geometry thereby limiting the ability of Ni^II^ to distort the site into its preferred planar geometry [5]. YeiR is analogous to CobW but is implicated in the delivery of Zn^II^ rather than Co^II^ [5, 16, 17]. In common with CobW, when bound to Mg^II^GTP, YeiR forms tighter complexes with Cu^I^ than with its cognate metal. How do the correct metals somehow partition onto these, and other, delivery pathways inside cells to avoid mis‐metalation, or blocked metalation, of the proteins that they supply?

### Example of kinetic trapping in a folded protein

Some metals become kinetically trapped within folded proteins. However, the folding pathway may still preferentially entrap the wrong metals. This is illustrated by the Mn^II^ cupin MncA. An experiment in which MncA was folded *in vitro* in the presence of an equimolar surplus of Cu^I^ and Mn^II^ led to the wrong metal, Cu^I^, being entrapped implying that Cu^I^ binds MncA more than 10 times more tightly. Similar experiments competing Mn^II^ against either Zn^II^ or Cu^II^ again led to entrapment of the wrong metal [4]. Presumably, some nascent flexible site along the folding pathway tends to follow the Irving–Williams series. Competition experiments with increasing molar excesses of Mn^II^ versus either Cu^II^ or Zn^II^ yielded full loading of MncA with Mn^II^ at 10 000 and 100 000‐fold surpluses of Mn^II^ respectively [4]. Notably, this suggests a slightly tighter affinity for Zn^II^ than Cu^II^, but the experiment used Tris buffers, which may have formed unaccounted Cu^II^‐complexes. Affinities for Zn^II^ and Cu^II^ binding to a nascent site in MncA have been assigned in Fig. 1D to reflect the measured differences relative to Mn^II^.

### Mis‐metalation within cells

The Mn^II^ form of *Escherichia coli* superoxide dismutase (SodA) is commonly mis‐metalated with Fe^II^ and inactive [18]. The detection of reactive oxygen species by a sensor, OxyR, triggers expression of a manganese importer (MntH) which in turn leads to nascent superoxide dismutase SodA being correctly metalated with Mn^II^ [19]. Small molecule cofactors can also become mis‐metalated. For example, exposure to elevated levels of Co^II^ leads to mis‐metalation of iron sulfur clusters with Co^II^ [20]. Furthermore, some forms of iron deficiency cause the accumulation of zinc protoporphyrin IX in place of haeme [21]. The copper cupin CucA is found in the same cyanobacterial periplasmic compartments as Mn^II^MncA, but CucA does acquire copper using the same ligands within the same fold as MncA [4]. Importantly, while CucA is secreted *via* the sec‐pathway to fold within the periplasm where it acquires copper, MncA is a TAT‐substrate which folds in the cytoplasm where it entraps Mn^II^. This suggests that the location of protein folding can determine the specificity of metalation, and moreover that Mn^II^ must be significantly more available than either Cu^I^ or Zn^II^ in the cytosol, with the latter ratio being at least 100 000‐fold. These observations reveal the crucial contributions of metal availabilities at the sites of protein folding to the avoidance of mis‐metalation [4].

## Metalation in cells

Cells have a diversity of mechanisms that maintain metal availabilities within tolerable ranges [22, 23, 24, 25, 26, 27, 28, 29, 30]. For example, importers acquire more of metals that are deficient while storage proteins and exporters sequester or remove those in surplus. Mechanisms also exist to sustain optimal metal availabilities within intracellular compartments and to maintain extracellular systemic metal supply in multicellular organisms. These mechanisms are controlled by a variety of metal sensors. DNA‐binding metal sensing transcriptional regulators have been especially well characterised in bacterial cells: They include metal‐dependent de‐repressors [31], metal‐dependent co‐repressors [32], and metal‐dependent activators [33, 34], of gene transcription. The allosteric mechanisms of these sensors have evolved to couple metal binding to DNA binding, and to respond within the ranges of intracellular metal availabilities over which their cognate metals fluctuate in viable cells [35, 36]. When sensitivities are adjusted to lie outside the vital range, sensors become unresponsive to changes in metal levels [35]. These sensors offer a route to read‐out the ranges over which intracellular metal availabilities fluctuate.

### Calibrating metal sensors to decode metalation

A set of DNA‐binding metal sensors of the three types outlined above, have been characterised in detail from *Salmonella enterica* serovar Typhimurium (hereafter *Salmonella*), for the purpose of calibrating their responses to intracellular metal availabilities [3]. The *Salmonella* sensors are almost identical to those of *E. coli* [5, 37]: They include Mn^II^‐responsive co‐repressor MntR, Fe^II^‐responsive co‐repressor Fur, Co^II^‐responsive de‐repressor RcnR, Ni^II^‐responsive co‐repressor NikR, Zn^II^‐responsive activator ZntR, Zn^II^‐responsive co‐repressor ZntR and Cu^I^‐responsive activator CueR [3]. After confirming their cognate metals, the metal affinities, DNA affinities of apo‐ and metal‐bound forms of each sensor, along with the number of promoter binding sites and the number of sensor molecules per cell (in high and in low metal) were all determined [3]. These values were used to calculate response curves that relate the state of each sensor to the intracellular availability of the cognate metal. The states (on or off DNA, with or without metal) that exist at different metal availabilities form coupled thermodynamic cycles [38, 39]. Before resolving these cycles mathematically in order to generate response curves, there needs to be prior consideration of the nature of available metals inside cells.

A Zn^II^‐buffered *in vitro* transcription assay previously established that Zn^II^ sensors of *E. coli* respond at femtomolar Zn^II^ concentrations [40]. It was noted that one atom per cell volume (approximately a femtolitre) equates to nanomolar concentrations, suggesting that the sensors detect the transition from Zn^II^ deficiency to excess at a million times less than one (hydrated) atom per cell. An explanation is that there are surplus binding sites in the intracellular milieu such that all Zn^II^ atoms are bound, leading to a suggestion that Zn^II^ might be delivered to its destinations by dedicated proteins analogous to copper metallochaperones [40, 41]. There is evidence of some Zn^II^ delivery proteins [42], albeit this deflects attention towards the specificity of metal acquisition by the delivery proteins themselves. Moreover, it is anticipated that Zn^II^‐metallochaperones are exceptional, with a multitude of Zn^II^‐proteins acquiring metal directly. Importantly, metal transfer by associative ligand exchange, analogous to transfer from metallochaperones [13, 41, 43, 44], may also occur from small molecule ligands such as glutathione or free histidine [35, 45, 46, 47, 48, 49, 50, 51, 52].

Cytosolic metal buffering by small molecules, metallochaperones and other labile sites, coupled with associative metal‐transfer, has several implications: (a) It becomes mathematically possible to resolve the coupled thermodynamic cycles to calculate sensor states at different metal availabilities when the metal is buffered (because metal binding to the sensor does not alter the available metal concentration, thus removing an otherwise dependent variable); (b) metal transfer by associative ligand exchange is rapid because it is not limited by the slow rate of release to the hydrated state; (c) an equilibrium state will better approximate an *in vivo* state if metal transfer to and from sensors (along with other proteins) is associative and fast; (d) an almost non‐existent pool of hydrated metal ions is not a limitation if metal transfer is associative; (e) the concentration of the negligible hydrated pool (equating to one Zn^II^ atom per million cells at any given instant or an atom in every cell one millionth of the time, in the earlier example) enables the strength of competition from the intracellular milieu to be calculated as an activity or difference in free energy. The range of internal metal availabilities over which each *Salmonella* sensor transitions between its off‐DNA states and its DNA‐bound states, or in the case of the activators, DNA‐metal‐bound state, were thus calculated [3]. Notably, the activators distort DNA to align critical nucleotide sequences only in their metal‐bound state. In contrast, repression is mediated by apo‐ or metalated sensors, albeit the proportions associated with DNA differ for co‐repressors versus de‐repressors. The calculated response curves were seen to depart from those generated from the metal affinities of sensors alone [3]. For example, some metal sensors are autoregulatory and a change in sensor abundance with metal concentration introduces hysteresis. Also, because metal binding and DNA binding are allosterically coupled, metal binding alters DNA affinity but reciprocally DNA binding alters metal affinity, and this influences the metal response curves.

### Metal availability follows the inverse of the Irving–Williams series

**Fig. 2:**
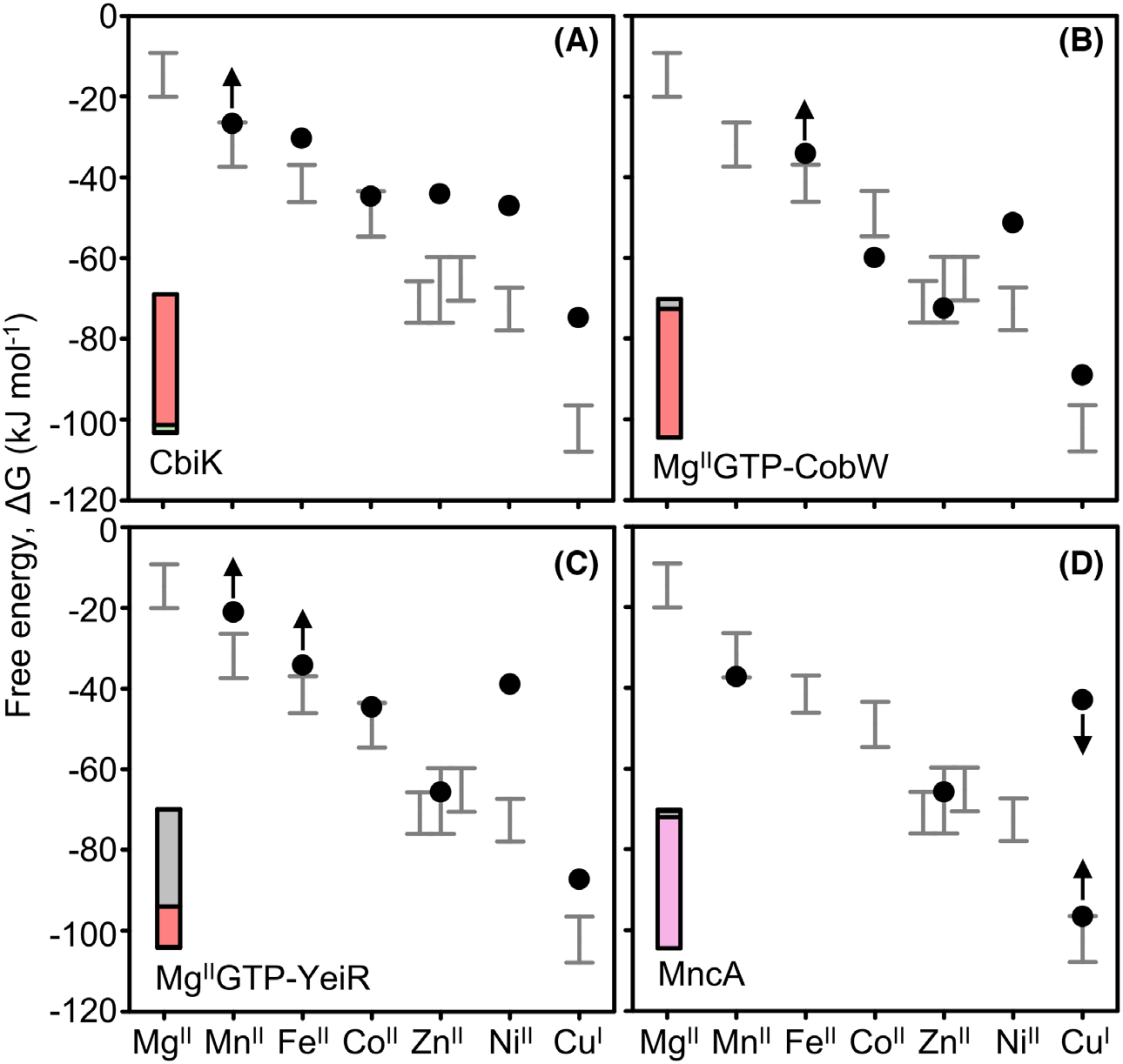
Metal availability is the inverse of the Irving–Williams series and decodes correct metalation. Grey bars show the ranges from 10% to 90% of the transcriptional responses of the cognate sensors for each metal as free energies for forming complexes that would be 50% saturated at the respective availability [3]. Metal availability is inverse to the Irving–Williams series. The ranges indicate the range of strengths of competition from exchangeable cytosolic binding sites against which the sensors have evolved to compete to sustain optimal metal availabilities. Metalation of other proteins similarly involves competition with these exchangeable metal binding sites. Black circles and arrows replicate metal binding data from Fig. 1, except for limits to Cu^I^ binding to MncA where the weakest value is derived from a competition experiment and the tightest inferred to give negligible (1%) Cu^I^ occupancy. The four cognate metals become apparent when binding is considered in relation to availability, as shown in the insets with Co^II^ (salmon red), Zn^II^ (grey), Mn^II^ (pink) [3, 5]. These proportional metal occupancies are calculated for idealised cells in which the sensors are at the mid‐points of their ranges. Total calculated metal occupancies are 16% CbiK, 99% Mg^II^GTP‐CobW, 36% Mg^II^GTP‐YeiR and (using the selected *K*_A_ Mn^II^) 91% MncA, implying substantial amounts of apo‐CbiK and apo‐YeiR exist under these conditions. Online metalation calculators similarly decode metal occupancies in the context of defined metal availabilities (https://mib‐nibb.webspace.durham.ac.uk/metalation‐calculators/) [53].

The grey bars in each panel of Fig. 2 show the availabilities over which the sensor(s) for each metal are calculated to respond, ranging from 10% to 90% cognate DNA occupancy with sensor, or with solely metalated sensor for activators [3]. Availabilities are shown as free energies for forming complexes that would be 50% metalated at the respective metal concentration. Metal availability is the inverse of the Irving–Williams series. The more competitive metals are maintained at the lowest availabilities. This has been a long‐standing hypothesis which is now experimentally supported by the calculated sensitivities of a cells' detectors/controllers of metal availabilities [3, 6]. These data provide estimates of the magnitude of competition from labile binding sites in the intracellular milieu [3]. In turn, this provides a frame of reference against which it becomes possible to re‐interpret metalation, for example, of the four proteins shown in Fig. 1, in a biological context.

### Decoding metalation

The insets in Figs 1 and 2 depict fractional occupancies of each protein with metals: copper black, Zn^II^ grey, Co^II^ salmon red, Fe^II^ green and Mn^II^ pink. Once competition against the intracellular milieu is considered, calculated metalation switches from the black and grey insets in Fig. 1 (representing the tightest but wrong metals copper and Zn^II^) to the colours in Fig. 2 [representing the correct metals Co^II^ (Mg^II^GTP‐CobW), Zn^II^ (Mg^II^GTP‐YeiR), Co^II^ (CbiK) and Mn^II^ (MncA)]. These occupancies have been estimated for an idealised cell in which metal‐availabilities match the mid‐points of the ranges for the respective sensor(s) [3, 4, 5]. They have been calculated from the difference in free energy for forming the respective metal complex with the protein of interest, versus that inferred for the competing intracellular milieu. Metalation can occur if the gradient favours transfer to the protein [3, 5]. The gradient may be favourable for more than one metal and hence values have been inter‐competed to ensure that occupancy of a single site does not exceed a stoichiometry of one [5]. Intriguingly, in the absence of nucleotide or with Mg^II^GDP, the gradient disfavours transfer of Co^II^ to CobW, whereas Mg^II^GTP‐CobW favours Co^II^‐transfer, providing insight into the mechanistic cycle for this metallochaperone [5]. Because metals are trapped within MncA it is difficult to determine the affinities of the nascent site at which binding occurs during folding and, as noted earlier, values have only been estimated for the relative affinities of three metals [4]. Fig. 2D therefore, shows the free energies for forming Zn^II^ complexes (and limits for Cu^I^ complexes) with the nascent site in MncA relative to an assigned Mn^II^ value.

Metalation calculators have been created which perform analogous calculations. They enable simulations of intracellular metalation of proteins of interest from inputted metal affinities and either by using default metal availabilities originally estimated for *Salmonella*, or by inputting known or simulated metal availabilities for other organisms (such as *E. coli*) [53].

## New frontiers in protein metalation

### Questions and methods

To what extent does metal‐protein speciation depart from predictions based on differences in free energies for complex formation relative to competing intracellular sites? Constraints and uses of the approach described here have been catalogued [53]. Additional factors that could influence metalation include kinetic contributions such as proximity to sites of metal import (where availability departs from that averaged over the compartment as a whole) and selective interactions with metal buffering molecules, including metallochaperones as extreme examples. The scale of such additional contributions could become evident from the extent to which observed metalation departs from the predictions of metalation calculators. The disparities may be relatively small, and hence at risk of being dismissed, but viewed in the context of the landscape of competition from intracellular‐binding sites their crucial contributions to correct metalation might become evident.

To what extent do the metal‐binding preferences of some proteins depart from the Irving–Williams series? A cautionary note is that metal affinities of proteins can be challenging to measure and many reported values are not correct [54, 55]. We have already discussed how the formation of adducts with other molecules such as Mg^II^GTP can pre‐organise a binding site to introduce steric selection [5]. It is known that cooperativity at di‐metal sites can similarly improve selectivity, for example, in favour of Mn^II^ relative to Fe^II^ in a class Ib diMn^II^ ribonucleotide reductase and in the Mn^II^/Fe^II^ oxidase R2lox [56, 57]. Change in oxidation state post‐binding, away from that of the labile pool, can favour retention of a selected metal [58]. Synthetic proteins have been generated with metal preferences that depart from the Irving–Williams series [59]. If better metal selectivity could have evolved, why has not it? Perhaps, because greater selectivity comes at a price such as reduced flexibility at the active site diminishing the catalytic repertoire [57, 59]. Perhaps because there has been limited pressure for greater selectivity when evolution has occurred within the thermodynamic landscape for metal availabilities shown in Fig. 2. Evolution of metal homeostasis, rather than adaptation of the vast complement of metal sites, has probably offered the more parsimonious solution when metal supply has changed over time.

By how much does metal availability vary in different compartments and organisms? It is anticipated that availability is the inverse of the Irving–Williams series in the compartments of most cells (e.g. albeit Cu^I^ may be substantially more available in the trans‐Golgi network of eukaryotic cells). Existing metalation calculators could initially be extrapolated to simulations for other cell types. However, modest change in availabilities of two metals, but in opposing directions (one more available, one less available), could switch the specificity of metalation. Thus, bespoke calculators should ideally be generated by substituting availabilities determined for the respective compartment and growth condition, albeit using the same understandings and web‐based template. However, it took about a decade to calibrate the sensors of *Salmonella* and in many compartments and species it is less clear which cellular sensors could be used to replicate this approach. The metal affinities of sites that modulate the trafficking or processing of metal‐transporters, change the stability or translatability of transcripts encoding proteins of metal homeostasis, or modulate other post‐transcriptional mechanisms, offer a possible route to define metal availability in idealised cellular compartments. However, it is less clear how these sites could be used to read out availabilities in conditional cells. An attractive idea is to use artificial intelligence to predict availabilities based on global surveys of protein metal affinities. However, a preponderance of erroneous affinities in the literature could confound the signal to noise ratio. Better yet, metal‐responsive probes, including cell permeable chromogenic molecules, have been developed [60, 61, 62]. There is uncertainty about what some probes read‐out in a biological context: But these uncertainties seem resolvable such that the probes could be calibrated to read‐out the free energies of available metal. Furthermore, these probes could be cross‐correlated inside *Salmonella* by comparison with values obtained from the characterised DNA‐binding metal sensors. This latter approach may allow the generation of bespoke metalation calculators for a variety of cells and compartments to be more swiftly created.

### Applications

The term nutritional immunity encompasses mechanisms by which pathogens are subjected to metal excess or deficiency as part of the host defences [23, 30, 63, 64]. This includes the sequestration of metals by calprotectin released from neutrophils [65, 66], the depletion of metals in macrophage phagosomes by natural resistance‐associated macrophage protein one [67, 68], the elevation of copper in the same compartment [69], the sequestration of iron scavenging siderophores by siderocalins [70], amongst others. There is a history of using metals and chelants to limit the growth of pathogens in medicine, in agriculture and in consumer goods [71]. Knowledge of the activities of available metals in pathogens should enable the identification of proteins that are liable to mis‐metalation, creating opportunities to tailor antimicrobials to subvert metalation, for example of enzymes involved in antimicrobial resistance.

Mis‐metalation occurs in some diseases [72]. This might be a primary cause or a secondary symptom. Metalation could be simulated for proteins associated with such diseases to identify those liable to mis‐metalation, perhaps informing future treatments. Such simulations could use metalation calculators in which availabilities have been entered that match determined free energies of available metals in the respective compartment of human cells. The latter might be determined using cell permeable chromogenic probes as discussed earlier. In plants, the generation of analogous calculators for their varied compartments has the potential to assist approaches to improve the nutritional supply of metals associated with hidden hunger [73].

The sources of some technology‐critical metals required in electronic devices and batteries are at risk [74]. This generates a need for targeted metal recovery and sustainable recycling. Sensors are known that detect several non‐essential metals and there is scope to identify more [33, 34, 75, 76, 77, 78]. These sensors could be calibrated to monitor and quantify sub‐lethal intracellular availabilities of the critical elements. In turn, this knowledge would assist the engineering of accumulation and bio‐recovery of technology‐critical metals.

In synthetic biology, heterologous (introduced) proteins may be mis‐matched to metal availabilities in the engineered cells. There is scope to use metalation calculators for organisms such as *E. coli*, and in future yeast, to optimise metalation in support of sustainable industrial biotechnology. The heterologous proteins might be products of *in vitro* evolution or of targeted engineering. Encouragingly, these proteins need not be engineered for the tightest binding metal to be the correct metal, but merely to meet the more attainable goal of acquiring the correct metal in the context of the prevailing intracellular availabilities as illustrated in Fig. 2. The pathway for synthesis of cofactor F_430_ stalled at the point of Ni^II^ insertion; likewise, that for vitamin B_12_ stalled at Co^II^ insertion, when introduced into *E. coli*, which does not naturally produce either molecule [5, 79]. The latter vitamin B_12_ pathway involved CobW as in Figs 1B and 2B. Calibrated qPCR with *E. coli* transcripts indicated that intracellular Co^II^ availability in cells grown in LB medium was below 10% of the range for Co^II^‐sensing RcnR, while Zn^II^ approximated to the mid‐point for Zn^II^‐sensing ZntR and Zur, equating to idealised cells for Zn^II^ but not for Co^II^ [5]. Under these conditions, Mg^II^GTP‐CobW is predicted to be ~75% mis‐metalated with Zn^II^ [5]. Supplementation of culture media with 10 μm cobalt was estimated (*via* qPCR) to raise the intracellular free energy of available Co^II^ sufficiently to reverse mis‐metalation of the metallochaperone and indeed under these conditions, vitamin B_12_ synthesis proceeded, matching calculated loading of Mg^II^GTP‐CobW with Co^II^ [5]. This presents opportunities to optimise the bioprocess for manufacturing vitamin B_12_ by manipulating the supply of Co^II^ or Zn^II^ *via* supplementation and chelation or by further engineering metal homeostasis. Importantly, vitamin B_12_ is neither made nor used by plants with the vegan society recommending supplements [80, 81]. As individuals adopt more plant‐based diets to reduce environmental demand for food production, efficient bio‐manufacture of vitamin B_12_ may gain in importance. Web‐based metalation calculators are now available for *E. coli* strains grown under specified culture conditions with plans to iteratively update the resource (https://mib‐nibb.webspace.durham.ac.uk/metalation‐calculators/) [53]. With an estimated half of the reactions of life requiring metals, optimisation of metalation informed by metalation calculators, promises to assist the transition to more sustainable manufacturing.

## Data Availability

Data sharing is not applicable as no new data were created or analysed in this review.
